# Altering Compositional Properties of Viral Genomes to Design Live-Attenuated Vaccines

**DOI:** 10.3389/fmicb.2021.676582

**Published:** 2021-06-30

**Authors:** Marianoel Pereira-Gómez, Lucía Carrau, Álvaro Fajardo, Pilar Moreno, Gonzalo Moratorio

**Affiliations:** ^1^Laboratorio de Virología Molecular, Centro de Investigaciones Nucleares, Facultad de Ciencias, Universidad de la República, Montevideo, Uruguay; ^2^Laboratorio de Evolución Experimental de Virus, Institut Pasteur de Montevideo, Montevideo, Uruguay; ^3^Department of Microbiology, Icahn School of Medicine at Mount Sinai, New York, NY, United States

**Keywords:** RNA viruses, genome composition, codon usage, codon pair bias, mutational robustness, attenuation, vaccines

## Abstract

Live-attenuated vaccines have been historically used to successfully prevent numerous diseases caused by a broad variety of RNA viruses due to their ability to elicit strong and perdurable immune-protective responses. In recent years, various strategies have been explored to achieve viral attenuation by rational genetic design rather than using classic and empirical approaches, based on successive passages in cell culture. A deeper understanding of evolutionary implications of distinct viral genomic compositional aspects, as well as substantial advances in synthetic biology technologies, have provided a framework to achieve new viral attenuation strategies. Herein, we will discuss different approaches that are currently applied to modify compositional features of viruses in order to develop novel live-attenuated vaccines.

## Introduction

Recent technological advancements in the field of synthetic biology have enabled the design of synthetic viruses with customized genome. Moreover, affordable DNA synthesis combined with reverse genetics improved the infrastructure to develop live-attenuated vaccines (LAVs). In the near future, synthetic RNA technology will also play an important role in reducing laboratory time and technical steps (Abil et al., 2015; Martínez et al., 2016, 2019; Pardi et al., 2018). The main goal of a LAV is to obtain a virus that does not cause disease but elicits a strong protective immune response against viral diseases. Classically, the generation of attenuated viruses is achieved by passaging the virus in tissue culture under different conditions (e.g., different host species or low temperature), which accumulates mutations supporting viral adaptation to the specific condition (Minor, 2015). As a result, several of these mutations will have a fitness cost in the original host or growth under physiological conditions and thus provide the basis for attenuation. However, the exact mechanisms by which these mutations lead to attenuated phenotypes are usually poorly characterized (Lauring et al., 2010). Despite their great capacity to induce a protective immune response, LAVs have the potential to revert to virulent phenotype by both reversions and/or introduction of compensatory mutations or by recombination with similar viruses (Cann et al., 1984; Kew et al., 2002; Bull et al., 2018). This is of special relevance to RNA viruses, due to their high mutation rates and rapid evolution (Duffy et al., 2008; Sanjuán et al., 2010). In order to overcome the risks presented by traditional LAV development approaches, several methods based on reverse genetics and genome recoding strategies have been developed. For instance, synthetic genome recoding approaches are based on introducing synonymous mutations into a protein coding region without modifying the protein sequence. The goal behind these strategies is that recoded viruses can replicate efficiently *in vitro* but can have limited or absent virulence *in vivo*, allowing the host to adopt effective immune responses with minimal risks of disease (Wimmer et al., 2009; Osterrieder and Kunec, 2018). It is worth noting that although numerous point mutations are introduced, the amino acid sequence of the parental virus and, therefore, its antigenic properties are preserved in the genome recoding strategies. In this review, we will focus on the fundamental role that genome composition has on RNA virus biology and evolution to design different attenuation strategies.

## The Relevance of Genome Composition

Genome composition is a highly variable trait that has a significant impact on the overall biology of organisms. Variability in genome composition can be readily observed by looking into codon usage and codon pair bias. For each species, a preferred set of codons is more frequently used than other synonymous ones that could perform the same role. Also, certain codons tend to be found next to each other more frequently than others, while some codon pair combinations are found infrequently. These results in codon usage or codon pair biases. Compelling evidence suggests that one of the key factors explaining codon usage bias in organisms, including viruses, is their specific nucleotide composition (Knight et al., 2001; Chen et al., 2004; Palidwor et al., 2010; van Hemert et al., 2016; Novoa et al., 2019). In RNA viruses, nucleotide composition is highly influenced by mutational pressure, referring to a bias toward, or away from, certain types of nucleotide substitutions or mutations (Jenkins et al., 2002; Seronello et al., 2011). In fact, studies have shown that mutational pressure or uneven base composition accounts for much of codon usage in RNA viruses (Woelk and Holmes, 2002; Seronello et al., 2011).

Mutational pressure will also determine the genome GC content, referred to as the percentage of guanines and cytosines in a given DNA or RNA sequence, which is also believed to be an important factor shaping codon usage (Sharp et al., 1993; Belalov and Lukashev, 2013). In addition, dinucleotide frequencies – that is, the occurrence of two nucleotides together in a DNA or RNA sequence – can also have significant impact on codon usage or codon pair biases. For instance, CpG dinucleotides are underrepresented in small DNA viruses and most vertebrate RNA viruses, including retroviruses (Karlin et al., 1994; Rima and McFerran, 1997; Shackelton et al., 2006). This CpG restriction is beneficial because it allows the evasion of the host innate immune responses by several pattern recognition receptors. UpA dinucleotide frequency is also strongly underrepresented among both RNA viruses and their corresponding hosts mRNA (Simmonds et al., 2013). This dinucleotide pattern could be explained by the presence of TpA in two out of three stop codons, thus decreasing the probability of generating non-sense mutations (Lobo et al., 2009). Also, some RNA-degrading enzymes involved in RNA stability and protein expression target UpA dinucleotides and present additional explanations for UpA bias in organisms (Beutler et al., 1989; Duan and Antezana, 2003; Simmonds et al., 2013).

Some other factors like RNA secondary structure, viral genomic organization, and life cycle may also contribute to codon bias in RNA viruses: for example, segmented viruses have stronger codon bias than non-segmented ones, and vector-borne viruses have lower codon bias than other viruses, likely due to their need to replicate in disparate hosts (Jenkins and Holmes, 2003).

Other explanations for codon usage bias proposes that as viruses depend on the host cell machinery for their own replication, genome composition and codon biases could reflect their host (Lobo et al., 2009; Simón et al., 2017). For instance, a virus may avoid using less abundant tRNAs present in the host to avoid slower rates of translation (Cattaneo et al., 1988; Hajjar and Linial, 1995). In this sense, it has been observed that some viruses seem to mimic the codon usage of their host (Carbone, 2008; Bahir et al., 2009; Su et al., 2009), but also others show different codon preferences (Gu et al., 2004; Sau et al., 2005, 2006). In addition, some codon pairs deviate significantly from their expected frequency (Moura et al., 2005), which seems to influence elongation rates during translation (Irwin et al., 1995).

Genome composition also plays a very important role in defining the mutational robustness of organisms, which refers to the capacity to withstand mutations, showing little or no phenotypic variation when a mutation is introduced (de Visser et al., 2003; Wagner, 2005). Mutational robustness is intrinsically linked to codon usage, since synonymous codons will code for the same amino acid, but they can differ in their evolutionary potential or evolutionary trajectories after single mutations, the most common type of mutations in RNA viruses. The evolutionary potential for any codon within a group of synonymous codons will be determined by two main factors: the base composition and the mutation itself. The nucleotide composition for a given codon is fixed and defines its coding capacity. Also, it will define the genetic relation or proximity to other neighboring synonymous or non-synonymous codons. On the other hand, mutation is a random trait that will have different impacts based on the type of point mutation (transition or transversion) and position (first, second, or third position) where the mutation is introduced. As a result, after a point mutation, different synonymous codons will present different likelihoods of remaining synonymous or, on the contrary, mutating into a farther away non-synonymous codon based on these factors. Thus, the genome composition can directly impact mutational robustness.

Strategies to attenuate viruses mostly based on genome recoding have been studied and reported elsewhere (Martínez et al., 2016, 2019; Gonçalves-Carneiro and Bieniasz, 2021). Here, we will summarize reported genome recoding strategies for viral attenuation from an evolutionary standpoint.

### Rational Alteration of Dinucleotide Frequencies to Achieve Viral Attenuation

As previously stated, vertebrate RNA viruses tend to exhibit underrepresentation or overrepresentation of dinucleotide frequencies (Rima and McFerran, 1997). In particular, UpA and CpG frequencies are consistently underrepresented across RNA viruses. It has been proposed that CpG restriction is beneficial because it allows the evasion of the host innate immune responses via several pattern recognition receptors including retinoic acid-inducible gene-I-like receptors (RIG-I-like receptors), mitochondrial antiviral signaling proteins (MAVS), protein kinase R (PKR), and others (Atkinson et al., 2014; Tulloch et al., 2014). More specifically, CpG underrepresentation has been associated with poor recognition via the zinc finger antiviral protein (ZAP). This protein, which can be ubiquitously expressed or induced the type I interferon (IFN), specifically detects viral RNAs that have a higher frequency of CpG dinucleotides compared to host mRNAs (Meagher et al., 2019) and promotes RNA degradation by endonucleases or RNA exosomes (Gao et al., 2002; Bick et al., 2003; Müller et al., 2007; Takata et al., 2017; Ficarelli et al., 2019; Luo et al., 2020).

**FIGURE 1:**
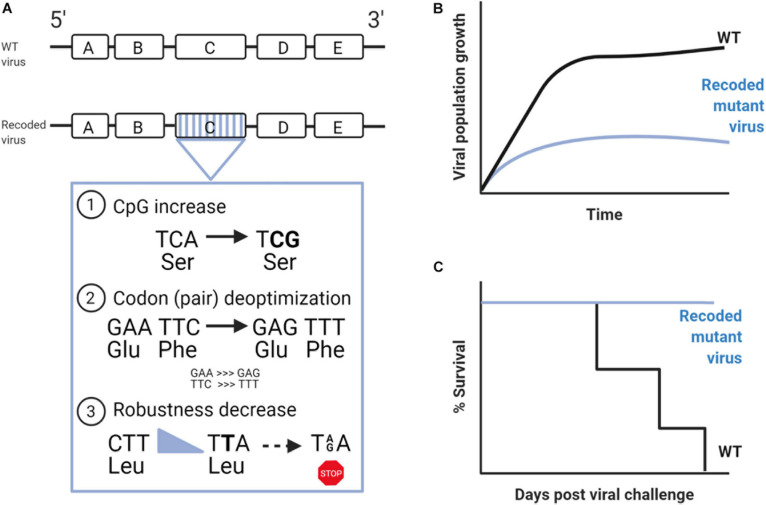
Strategies to rationally attenuate viruses and develop live-attenuated vaccines. **(A)** Scheme of viral genomes; above is the wild-type (WT) genome, and below is indicated the recoded viral genomes by the main approaches used to attenuate viruses. **(B)** Viral population growth and **(C)** survival curves expected from a recoded virus and the WT.

Extensive evidence has indicated that enriching viral genomes with CpGs results in a loss of fitness and could therefore provide an alternative strategy to achieve viral attenuation (Figure 1; Burns et al., 2009; Tulloch et al., 2014; Gaunt et al., 2016; Takata et al., 2017). Burns et al. (2009) directly approached this by introducing random sets of synonymous codons into the capsid coding region of the poliovirus genome. *In vitro* studies with these mutant viruses revealed that only those with increased CpG and UpA frequencies had fitness loss, and this was due to decreased viral infectivity (lower ratio of infectious particle per genome copy number) and not by decreased translation efficiency (Burns et al., 2009). Later, this inverse relationship between dinucleotide frequencies and viral fitness was confirmed in a study on echovirus 7 in which a control virus with decreased CpG and UpA frequencies showed an increase in viral fitness (Atkinson et al., 2014; Tulloch et al., 2014). Also, the authors proposed that the attenuation was mainly driven by enhanced response of the innate immune response. The first studies with recoded viruses in animal models were done with the influenza virus, showing that only the high-CpG virus (but not the high-UpA virus) was attenuated in mice. Moreover, mice infected with high-CpG viruses presented decreased clinical severity, good induction of the innate and adaptive immune responses, and reduced pathology in the lung. Thus, additional evidence to consider these viral constructs as vaccine candidates was provided (Gaunt et al., 2016).

### Attenuation by Deoptimization of Codons and Codon Pairs

The redundancy of the genetic code allows adjusting the efficiency in the production of proteins at different levels, without modifying the amino acid sequence (Gingold and Pilpel, 2011). Most amino acids are encoded by more than one codon, and the frequency at which these synonymous codons are used defines the codon usage. The preference for different codons has significant effects on mRNA stability (Presnyak et al., 2015), translation efficiency and accuracy (Gingold and Pilpel, 2011), and protein folding (Zhang et al., 2009).

It is assumed that codons that are used to a greater extent by cellular mRNAs correspond to more abundant tRNAs in the host cell (Ikemura, 1985; Plotkin and Kudla, 2011). Consequently, recoding viruses with infrequent host codons should reduce the translation rates and protein yield, leading to its attenuation (Figure 1; Presnyak et al., 2015). This approach was first implemented to recode the capsid protein of polioviruses, leading to reduced amounts of infectious progeny with a sharp decrease in their replicative fitness (Burns et al., 2006). However, the immunogenicity of these engineered viruses was conserved as they continue to elicit robust neutralizing antibody responses (Burns et al., 2006; Mueller et al., 2006). Subsequent studies were performed to rationally recode other picornaviruses (Diaz-San Segundo et al., 2016), as well as different negative stranded RNA viruses (Meng et al., 2014; Nogales et al., 2014; Cheng et al., 2015, 2017; Stobart et al., 2016), giving rise to highly attenuated viruses both *in vivo* and *in vitro*, with the exception of rabies virus, in which attenuation could not be achieved (Wirblich and Schnell, 2011).

Along with codon usage, codon pairs (or a combination of triplets) have been reported as major players influencing the modulation of translation (Brar, 2016). As mentioned before, in every species, certain codon combinations are observed more frequently than others that are preferably avoided, which is known as codon pair bias (Gutman and Hatfield, 1989). These features can be exploited to reach viral attenuation by recoding viral open reading frames using suboptimal combinations of codon pairs, without affecting codon bias or amino acid profiles (Coleman et al., 2008). This reshuffling approach was successfully implemented to attenuate, both *in vitro* and *in vivo*, a broad range of positive (Coleman et al., 2008; Martrus et al., 2013; Shen et al., 2015; Song et al., 2017; Li et al., 2018) and negative stranded RNA viruses (Yang et al., 2013; Broadbent et al., 2016; Le Nouën et al., 2019). Moreover, several vaccines were developed by means of this strategy (Mueller et al., 2010, 2020; Cheng et al., 2017; Tsai et al., 2019), including the recent CDX-005 intranasal COVID-19 vaccine candidate that is currently undergoing phase I trial in the United Kingdom (Lundstrom, 2020; Codagenix starts phase I trial for Covid-19 vaccine in U.K, 2021).

The mechanisms underlying attenuation by deoptimization of codon and codon pairs are still controversial. Although codon choice may have a direct effect on translational efficiency and mRNA stability, codon pair deoptimization strategies will inevitably increase the frequency of usually underrepresented CpGs (Tulloch et al., 2014). This is because, although in the process of shuffling codons the bias among them is maintained, new CpGs emerge as most underrepresented codon pairs contain this dinucleotide at their boundary (Atkinson et al., 2014; Kunec and Osterrieder, 2016). Therefore, many studies have suggested that the attenuation is achieved due to antiviral activity displayed by the recognition of CpG-abundant RNAs by ZAP. However, evidence provided by a recent study suggests that suboptimal codon pairs, rather than CpG increase, are responsible for the attenuation of influenza A virus as a consequence of the reduction in translational efficiency and mRNA stability (Groenke et al., 2020). While opposing, it is likely that attenuation is achieved by a combination of both effects, since usually they go hand in hand, and it is difficult to separate their contributions in experimental settings. Moreover, it is also possible that each has varying impacts on different viral families, and thus, no universal rules can be proposed.

### Attenuation by Decrease in Viral Mutational Robustness

**FIGURE 2:**
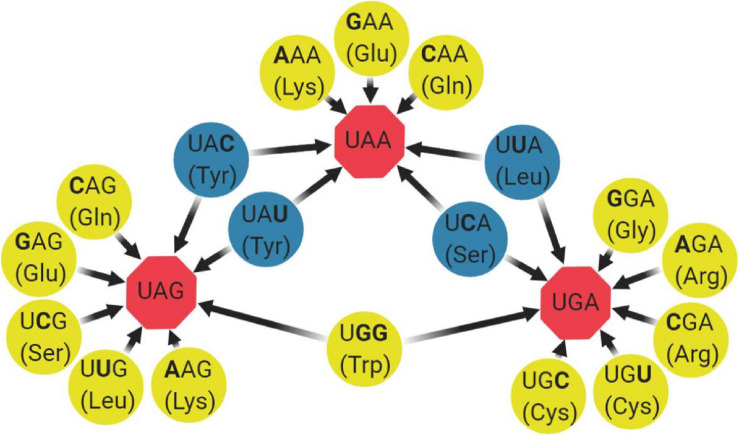
Codons containing non-sense mutation targets. From the 64 codons in the genetic code, 18 codons containing 19 different non-sense mutation targets can mutate to a stop codon after a single-base substitution (boldfaced). In most of the cases, only one of the three possible substitutions produces a stop codon (yellow circles), whereas in some scenarios, two of the three substitutions produce a stop codon (blue circles). Stop codons are represented by red octagons.

Robustness can be defined as phenotypic conservation in light of genetic variation. One of the most recent developments in the theme of attenuation by codon rearrangements involves decreasing the mutational robustness of RNA viruses (decreasing the capacity to “buffer” mutation effects). By rationally engineering viruses that are less able to “buffer” the burden of mutations, strong attenuation can be readily achieved (Figure 1; Lauring et al., 2012; Moratorio et al., 2017; Carrau et al., 2019). The first study of this kind was done in poliovirus, where synthetic viruses carrying reengineered capsid sequences with hundreds of synonymous mutations versus wild type were compared. The authors found that only the virus with decreased robustness was attenuated in mice and had also decreased in neurovirulence (Lauring et al., 2012). Years after, a study focusing only on synonymous codons with non-sense mutational targets (NSMTs) was proposed (Moratorio et al., 2017). By definition, NSMTs are those codon sites that can produce a non-sense mutation (stop codon) after a single-nucleotide substitution. From the 64 codon triplets, 18 codons containing 19 different non-sense mutation targets can mutate to a stop codon after a single-base substitution (Figure 2). Moreover, single-nucleotide substitutions are the most frequent type of spontaneous mutations in RNA viruses (Sanjuán et al., 2010), and the chance of two or three mutations landing in a single codon is extremely low. Based on these, the authors engineered viral genomes to have more serine and leucine codons with NSMTs, in regions of the Coxsackie virus B3 and influenza A virus, compared with wild-type viruses. The resulting increase in stop codon mutations during replication led to a loss of infectivity *in vitro* and attenuation *in vivo*. Moreover, attenuation was even stronger when they coupled it with a low-fidelity polymerase, since more stop codons were being generated during replication. Importantly, in this study, the authors were able to significantly remove the effect of confounding factors such as altering codon pair bias or dinucleotide frequencies and focus solely on the impact of rationally modifying mutational robustness. Next, a study by the same group applied this attenuation method on Chikungunya virus and expanded it by also incorporating codons for arginine and glycine with non-sense mutation targets. The proposed LAV was significantly attenuated in mosquito and mammalian hosts, had significantly reduced dissemination in mice, elicited good antibody responses that protected from challenge and had decreased transmissibility from mosquitoes to mice, thus being proven an efficient design (Carrau et al., 2019).

## Conclusion

Live-attenuated vaccines against viral diseases have been among the most successful medical developments in human history. However, these vaccines, which were generated by classical approaches, have occasionally shown cases of reversion, becoming unsafe and endangering human health. The most paradigmatic case is the oral polio vaccine that showed low genetic stability (Kew, 2012). This is in part because we have not yet fully elucidated the molecular basis of attenuation. In addition, we might underestimate the evolutionary potential of RNA viruses. In this way, the rational design of LAVs, considering the nucleotide composition of viral genomes and their evolution, becomes an important field for future development of LAVs.

Each of the genetic variations on the theme of codon rewiring mentioned in this review has been proposed as new methods for vaccine development or to improve safety of already-existing LAVs. Indeed, they represent good vaccine candidates because they have a strong attenuated phenotype yet exactly similar to wild-type viruses at the protein level, with native antigenicity and complete immunogenicity (Rima and McFerran, 1997; Coleman et al., 2008; Mueller et al., 2010; Cheng et al., 2017; Moratorio et al., 2017; Kaplan et al., 2018). Also, the method can be broadly applied, aided by computational tools that could recode any viral genome (Mueller et al., 2010; Jorge et al., 2015).

The main advantage over other LAV designs is that reversion to pathogenic phenotypes is very unlikely, since no single substitution is responsible for the majority of the attenuated phenotype. Rather, it is presumed that attenuation is the sum of dozens to hundreds of mutations, each imparting minor fitness costs – a strategy aptly described by its creators as “death by a thousand cuts” (Coleman et al., 2008). Nevertheless, if only a subset of codon substitutions has dominant effects, then the risk of reversion to a pathogenic form of virus would be higher than expected. Some *in vitro* studies have observed slight gain of fitness after extended passaging through reversion or compensatory mutations, but still within the range of the expected evolution for wild-type virus, hence not discouraging the use of these viruses as LAVs (Bull et al., 2012; Nougairede et al., 2013). Interestingly, in a study where the attenuation of a codon-deoptimized human RSV was lost, molecular dynamics simulations identified key positions that could restore the attenuation. When engineered back into the codon-deoptimized genome, a more attenuated virus was rescued with strong immunogenicity and increased stability, advocating for applied rational attenuation (Le Nouën et al., 2017). Even more, in the case of LAVs solely focused on reducing mutational robustness, reversion might seem even more unlikely, given that the introduced mutations do not interfere with replication or translation. If such was the case, selective pressures would be increased in order to restore fitness. Also, compensatory mutations would be less likely, because these viruses will be less able to explore sequence space, restricting the access to beneficial phenotypes.

By further elucidating each mechanism underlying these attenuated phenotypes, a rational evolutionary approach to codon rewiring could take advantage of combining increasing CpG and UpA frequencies to activate host innate immunity (Kumagai et al., 2008), forcing rare codons or codon pair bias to slow down translation (Chevance et al., 2014), and restricting a virus’ mutational robustness and/or detrimental mutational neighborhoods (Lauring et al., 2012; Moratorio et al., 2017; Carrau et al., 2019).

## Author Contributions

MP-G, LC, and ÁF reviewed literature and performed article conception, design, and writing of the manuscript. MP-G made the figures. PM critically revised the manuscript. GM performed article conception and drafting and critical revision of the manuscript. All authors read and approved the submitted version of the manuscript.

## Conflict of Interest

The authors declare that the research was conducted in the absence of any commercial or financial relationships that could be construed as a potential conflict of interest.
